# Closed Fracture Treatment in Adults, When is it Still Relevant?

**DOI:** 10.51894/001c.28060

**Published:** 2022-02-24

**Authors:** Matthew Coon, Marek Denisiuk, Derrek Woodbury, Benjamin Best, Rahul Vaidya

**Affiliations:** 1 Ascension Macomb-Oakland Hospital; 2 Orthopaedic Attending DMC Detroit Receiving Hospital; 3 Orthopaedic Attending Ascension St. John Hospital

**Keywords:** review, non-operative, closed treatment, trauma

## Abstract

**INTRODUCTION:**

Fracture treatment has been documented since the times of ancient Egyptian and Greek civilization, with fracture reduction techniques and the apparatus for immobilization developed over three millennia. Over the last 150 years, aseptic technique, anesthesia, antibiotics, and internal implants have changed how orthopedic specialists approach fracture care. More recently, there has been an increased promotion in the medical literature to evaluate the clinical outcomes of nonsurgical treatment of common upper and lower extremity closed fractures.

**METHODS:**

In this paper, the authors review the history of closed extremity fracture treatments, outline contemporary studies regarding treatments of non-displaced fractures, and discuss the recent literature that has informed orthopedic surgeon-patient decision-making discussions regarding closed fracture management.

**CONCLUSIONS:**

Based on the results of this literature review, orthopedic providers should consider the preferable outcomes associated with nonoperative fracture management such as lower infection rates, the possibility of rapid functional improvements and lower healthcare costs. Nonoperative methods for closed fractures can sometimes be more safely delivered even with more difficult fractures. This may be of particular benefit to patients with higher surgical risks, minimizing exposure to treatments that are not only more invasive and expensive, but that can impose greater postoperative risks.

## INTRODUCTION

Through the 20th century, the nonsurgical treatment of closed fractures (i.e., when bone is broken, but skin intact) have been the standard of care.^1^ The earliest documentation of fracture care was in the Egyptian “Edwin Smith” papyrus, circa 1600 b.c.^1^ Egypt was also the site of the earliest examples of active fracture care (e.g., splints) on an unhealed femur fracture, dated around 300 b.c.^2^

At approximately 400 b.c., Hippocrates wrote three books “Fractures,” “Articulations, and”Instruments of Reduction” for fracture management.^3^ He noted the five following principles of care: antisepsis, reduction, traction, bandaging, and splinting.^3^ In developing nations, “bonesetters” in Asia, Africa, and in native populations of North and South America have typically been non-medically trained practitioners treating fractures and reducing joint dislocations with skills developed using an apprentice model. Although lay bonesetters have not been accepted by many mainstream medical communities,^4^ present-day bonesetters in developing nations may still have their services preferred over modern medical techniques.^5^

Early methods to stabilize fractures recorded by Hippocrates included linen splints stiffened with gum and plaster; bandages suffused with resins, gums, and waxes.^3^ Bandages with lime and egg white have also been recorded by Arabic physicians.^6,7^ The use of plaster was first described in 1798 by British surgeons who had observed Persians using gypsum. In 1852, the Dutch military surgeon Matthysen devised a method to coat and infuse cotton bandages with gypsum to make the first casting bandages.^8^ During the 1930s, the addition of binders (e.g., starches, gums, and dextrins) made commercial bandage preparation more feasible, although it wasn’t until the mid-1940s that commercial plaster bandages became commonly used.^9^

Immobilization treatments of closed fractures (e.g., slings, splints, casts traction avoidance of weight bearing) are still the most widely used method of fracture management.^10^ Tables 1 and 2 depict the modes of immobilization, when to start range of motion, and when to return to normal function based on medical textbooks ^11,12^ as well as the senior authors’ (i.e., BB, DW, RV) clinical experiences. Due to subjective nature of considering closed fracture management options, it is recommended that each case be taken individually and tailored to the patient’s particular fracture pattern and morphology. Although these fracture patterns may initially be treated by an emergency medicine physician or other primary care provider, in the United States, these fractures are, as a standard of care, referred for fracture management to an orthopedic surgeon.

**Table 1.** Upper Extremity Closed Fracture Management Options

**Fracture**	**Treatment**	**Start Movement**	**Activity**	**Return to function**
Clavicle and Acromioclavicular Joint	Sling	Two-Three weeks	Two-Three weeks passive or active ROM*	Six-10 weeks
Proximal Humerus	Sling or airplane splint	Two-Three weeks	Two-Three weeks gentle ROM or active assisted six weeks active resistance	Six-10 weeks
Humerus shaft	Coaptation splint, sling and swath Two-Three weeks and switch to Sarmiento	Two-Three weeks pendulum / isometric	DC brace when active abduction painless and no fracture movement	12 weeks
Distal humerus, olecranon, coronoid, radial head	90-degree post mold cast or sling One-Two weeks	One-Two weeks	One-Two weeks passive ROM, active ROM Four-Six weeks when painless Strengthening at six weeks when painless	Six-12 weeks
Radius / Ulna	Splint 90 deg Switch to above elbow cast when comfortable	Four-Six weeks	Four-Six weeks ROM for elbow	Six-12 weeks
Distal radius / Carpus	Volar splint Switch to cast	Four-Six weeks	ROM active / Resistance exercises six weeks	Six-12 weeks
Metacarpals, Ulnar Two	Ulnar gutter splint	Three-Four weeks	ROM active / resistance exercises four weeks	Six-12 weeks
Metacarpals, Radial Two	Volar splint switch to cast	Three-Four weeks	ROM active / resistance exercises four weeks	Six-12 weeks

* ROM – range of motion

**Table 2.** Lower Extremity Closed Fracture Management Options

**Fracture**	**Treatment**	**Start Movement**	**Activity**	**Return to function**
Pelvic fracture	Protected weight bearing	Right away	Weight bearing when tolerated Two-Six weeks	Six-12 weeks
Acetabular fracture	No weight bearing on effected side	Right away	Weight bearing Six-12 weeks	12 weeks
Femur fractures, proximal and shaft, should not be treated nonoperatively				
Tibia fracture proximal	Above knee cast six weeks Hinged cast brace Six-10 weeks	Six weeks	Active ROM, weight bearing 10-12 weeks	12-20 weeks
Tibia shaft fracture	Above knee cast Four-Six weeks Sarmiento PTB* cast Four-12 weeks	Six weeks partial weight bearing	Active ROM, weight bearing 10-12 weeks	16-20 weeks
Lateral malleolar ankle fractures	Cast or fracture boot	ROM right away	Partial to full weight bearing as tolerated	12 weeks
Bimalleolar or equivalent fracture	Cast above knee two-four weeks Below knee up to six weeks	Four-Six weeks	Weight bearing Six weeks	12 weeks
Talus fracture	Cast or boot	Four-Six weeks	Weight bearing 12 weeks for neck or body fracture	16-20 weeks
Calcaneus fracture	Protective boot	ROM right away	Weight bearing Eight-12 weeks	16-24 weeks
Foot fractures	Protective boot or cast	ROM four weeks	Weight bearing Four-Six weeks	Six-10 weeks

* PTB – patellar tendon bearing

The following sections of this literature review will focus on the most common types of upper and lower extremity displaced fractures. Hand and foot fractures will not be reviewed. Classic and current studies will be discussed to provide readers a historical perspective and comprehensive review of the current state of non-operative treatment of displaced fractures in modern orthopedics.

## Displaced Upper Extremity Fracture Treatments

### Clavicle Fractures

Fractures of the clavicle account for up to 10% of adult fractures and up to 80% of these fractures occur in the middle third of the shaft.^13^ Non-displaced or minimally displaced fractures generally heal well with a sling for two to three weeks followed by physical therapy with a typical return to normal function in six to 10 weeks. Displaced clavicle fractures with higher-risk characteristics including 100% displacement, shortening greater than two cm., or Z-type (i.e., characterized by vertical positioning of a segmental fragment) fracture pattern have been reported to have a nonunion rate of up to 15% with nonoperative treatment (Table 3).^14^

**Table 3.** Clavicle Fracture Outcomes: Operative vs Nonoperative Treatment

Investigators	Sample Size	Findings	Miscellaneous
Amer et al., 2020	954 displaced (100%) or >2cm of shortening	Lower nonunion and symptomatic malunion with operative fixation; No difference in Constant or DASH* scores	Meta-analysis; operative vs nonoperative
Zlowodzki et al., 2005	2144 (97% midshaft)	Overall nonunion rate of 5.9% for nonoperative treatment; nonunion rate for displaced fractures is 15.1%	Systematic Review of 22 articles
Woltz et al., 2017	160 displaced midshaft	No difference in Constant and DASH scores; significantly higher nonunion rate in nonoperative group	Multicenter, RCT**; ORIF*** vs nonoperative treatment

** Randomized Controlled Trial

*** Open Reduction Internal Fixation

A 2017 Level I randomized controlled trial by Woltz et. al. compared open reduction internal fixation (ORIF) to nonoperative treatment for displaced midshaft clavicular fractures in adults. This group identified a higher rate of nonunion in the nonoperative group of 23.1% compared with 2.4% in the ORIF group. Despite a high union rate, operative treatment was also associated with a secondary surgery rate of 27.4% and peri-incisional anesthesia of 19%.^15^

### Scapula Fractures

Scapula fractures can be divided based on the energy (i.e., amount of force causing the fracture) involved and fracture location. Although traditionally considered high energy injuries (e.g. automobile accident), low energy injuries may occur in the elderly. In both groups, nonoperative management consists of sling immobilization with progression to physical therapy at two weeks. Consideration for ORIF should be made in cases involving glenohumeral instability, intra-articular involvement, and displacement.^16–18^

A 2018 systematic review of all scapular fracture types reported satisfactory results in 90.4% (N = 629) of nonoperatively treated patients and 93.7% (N = 512) of operatively treated patients.^19^ Nonoperative treatment for displaced scapular neck fractures > 10 mm demonstrated only 15.7% patient satisfaction, versus 94.7% satisfaction with displacement < 10 mm. Scapular body fractures treated nonoperatively resulted in excellent outcomes in 100% of patients. For coracoid (i.e., short, bony projection off scapula) fractures, nonoperative treatment led to excellent outcomes and is equivalent to surgical intervention. However, fractures of the scapular neck extending into the body resulted in satisfactory outcomes in only 50% of nonoperatively treated patients with surgery demonstrating superior outcomes.^19^

### Proximal Humeral Fractures

Since the 1950s, little has changed in the closed treatment of proximal humerus fractures. Nonoperative management typically consists of two weeks of sling application with a progressive physical therapy regimen for non-displaced fractures. Although the introduction of locked plating may have improved operative fixation of displaced fractures, several studies fail to show significant relative improvements in clinical outcomes (Table 4). Several systematic reviews and randomized studies ^20–24^ conducted between 2011 and 2019 comparing ORIF versus nonoperative treatment for three- and four-part fractures found no significant differences in Constant scores (i.e., four-variable scoring system used to assess the function of the shoulder) or other clinical outcomes at one year.

**Table 4.** Proximal Humerus Fracture Outcomes: Operative vs Nonoperative Treatments

Investigators	Sample Size	Findings	Miscellaneous
Mao et al., 2014	287 3- or 4-part	No difference in Constant, DASH, or total complication events	Meta-analysis; modest sample size
Fjalestad et al., 2012	48 displaced 3- or 4-part	No difference in functional outcome at 1-year follow up	RCT ORIF vs conservative
Lopiz et al., 2019	59 displaced 3- and 4- part	No difference in any patient-reported outcomes except VAS score at 12 months	RCT RSA vs conservative
Olerud et al., 2011	60 displaced 3- part	Indicate an advantage in functional outcome and HRQoL***** in favor of the locking plate	RCT ORIF vs conservative; 2 year outcome; 30% cost of additional surgery for surgical cohort
Rangan et al., 2015	250 displaced surgical neck	No difference in patient-reported outcomes over 2 years	RCT multicenter; internal fixation/replacement vs conservative

* DASH - Disabilities of the Arm, Shoulder and Hand

** RCT – randomized controlled trial

*** ORIF – open reduction internal fixation

**** VAS – visual analog scale

***** HRQoL - Health-related quality of life

### Humeral Diaphysis Fractures

Displaced humeral shaft fractures has been traditionally treated in nonoperative manner during substantial investigations confirming low nonunion rates and good outcomes.^25^ However, like many other displaced fracture patterns, a modern trend towards ORIF has been generating interest.^26^ Management typically involves initial treatment with a well-molded U-slab or coaptation splint with conversion to functional bracing at two weeks.^12^

The humerus easily tolerates 30 degrees of varus angulation and 20 degrees of anterior angulation which results in a functional range of motion of the upper extremity and normal cosmesis.^27^ Most patients in this and other studies have demonstrated good to excellent functional outcomes.^28–30^ The location of the fracture within the bone determines successful healing with union rates of up to 88% for middle and distal third shaft fractures and 76% for proximal third shaft fractures.^31^ However, certain humeral fracture characteristics (e.g., spiral and oblique fracture patterns) should guide orthopedic surgeons towards ORIF.^32,33^

### Forearm Fractures

Isolated ulna fractures can generally be treated with immobilization as long as there is some overlap of the fracture ends with proper alignment.^34^ Although stable, open ulnar fractures from both firearm and more severe non-firearm mechanisms may be treated conservatively if there are minimal osseous displacement and soft-tissue trauma, more severe open injuries are better managed with surgical ORIF.^35,36^

### Distal Radius Fractures

Many distal radius fractures can be reduced to the anatomic position of 12 mm. radial height, 11 degrees volar tilt, and 23 degrees radial inclination.^37^ Unfortunately, closed reductions may not retain their position over a two-to-three-week period until initial healing is obtained. This has been attributed to advanced age or the deforming forces caused by the surrounding musculature, resulting in longitudinal and angular deformities and fracture characteristics such as initial displacement and shortening.^38^

In 1989, Lafontaine described fracture characteristics that predicted a loss of reduction in cast immobilization if three or more of the following criteria were present: dorsal tilt >20 degrees, dorsal comminution, intra-articular fracture, associated ulnar fracture, and age over 60 years.^39^ The definition of fracture instability remains varied throughout the literature.^40,41^ The American Academy of Orthopedic Surgery Clinical practice guidelines advise operative fixation for fractures with post-reduction radial shortening of greater than 3.0 mm, dorsal tilt > 10 degrees, or intra-articular displacement or step-off greater than 2.0 mm.^42^ Comparative studies of operative versus nonoperative treatment of displaced distal radius fractures in elderly patients have shown better radiologic results, however, some studies have shown no advantage in functional outcomes (Table 5).^43–45^

**Table 5.** Distal Radius Fracture Outcomes: Operative vs Nonoperative Treatment

Investigators	Sample Size	Findings	Miscellaneous
Egol et al., 2010	90 displaced, unstable	Minor limitations in ROM of wrist and diminished grip strength with nonoperative care	Case-control study; Surgery vs conservative
Toon et al., 2017	60 closed, intra-articular	No difference in overall function at 12 months	Comparative study; ORIF vs conservative; Vast difference in treatment costs
Ochen et al., 2020	2254	No improvement in DASH scores in patients >60 yo	Meta-analysis; 8 RCTs and 15 observational studies

## Treatment of Displaced Lower Extremity Fractures

### Pelvic Ring Fractures

Low energy pelvic ring injuries tend to heal with time when treated conservatively.^46^ Indications for ORIF include high energy injuries, to prevent death from hemorrhage, deformity in displaced injuries, and to allow early mobility. Hemorrhage traditionally has been controlled with a pelvic binder, angiography, pelvic packing, and addressing non-pelvic causes of bleeding.^12^

For patients who are unable to sit or choose non-operative intervention, bed rest is recommended for one to two weeks followed by a gradual increase in activity over the next two to three months. Patients with displaced pelvic ring injuries greater than 1.0 cm who are treated conservatively tend to have a higher malunion rate,^47^ experience greater pain at longer-term follow-up, and have more difficulty ambulating compared to ORIF patients.^47^ In 2014, Gaski et. al. assessed the functional outcomes of potentially unstable lateral compression fractures, demonstrating that nonoperative treatment yielded acceptable functional and perceived health status outcomes in this population.^48^

### Femoral Shaft Fractures

Nonoperative treatment of femoral shaft fractures occurs in some developing nations, as well as in patients who are not amenable to operative treatment.^49^ One form of nonoperative treatment of femoral shaft fractures includes Perkins traction,^50^ which allows movement of the knee during traction. The results of Perkins traction are reported to have a nonunion rate of 10%, malunion rate of five percent, shortening > 2.5 cm, and acceptable range of motion. The average length of hospital stay is eight weeks, with a mean healing time approximately 10 weeks, and return to function about 16 weeks.^50–52^

Intramedullary nailing of femur fractures results in a 98% union rate, a low rate of leg length discrepancy (i.e., less than five percent; 20% in comminuted/splintered fractures), and rotational malalignment (i.e., as low as five percent). As a result, operative fixation of femur fractures with intramedullary nailing has become one of the great success stories of the 20th century for American orthopedic surgeons.^53^

### Tibial Shaft Fractures

Traditionally, tibial shaft fractures have been treated with traction (i.e., use of ropes, pulleys and weights to regain the original length of injured bone), casting, functional bracing, external fixation, plating, and intramedullary nailing.^54^ The treatment of choice for isolated, displaced closed tibia fractures has recently migrated to intramedullary nailing, secondary to high rates of union and low rates of malunion.^55^ Despite this shift, closed treatment remains a potentially viable option.^56,57^ Patients are typically placed in above knee long leg casts and switched to functional braces after three to five weeks.^58^ In Sarmiento’s 1989 series of 780 tibial fractures, the nonunion rate was 2.5%, shortening of < 10mm occurred in 90% of patients, and an acceptable angular deformity of < 10 degrees was generally attained.^58^

However, studies comparing intramedullary nailing to nonoperative treatment have demonstrated a decreased time to union, increased union rate, decreased malunion rate, early weight bearing, improved function, and earlier return to work and sports with surgery.^55^ During a 2020 retrospective study by Swat et. al., factors predictive of successful nonoperative treatment include initial coronal and sagittal translation, shortening, fracture morphology and location, body mass index, and smoking status.^59^

In a comparative study of casting versus intramedullary nailing of unilateral, displaced, isolated closed fractures of the tibial shaft, time to healing was shorter (i.e., 16 weeks vs. 18 weeks) and nonunion rates were reduced (two vs 10%) in the ORIF group. Functional outcomes were superior in the operative group.^55^

### Ankle Fractures

Ankle fractures are articular injuries of the mortise joint accounting for 9% of all adult fractures.^60^ Ankle fractures were historically treated with closed reduction and above knee casting for two to three weeks followed by below knee casting for three to four weeks or longer depending on healing potential.^61^ Currently, nonoperative treatment of ankle fractures includes a short leg cast, splint, or walking boot. Indications for closed treatment include rotationally stable fracture patterns where the syndesmosis remains intact, talus is reduced, and when an acceptable reduction is achieved. Conservative management of ankle fractures is a difficult task, frequently failing secondary to a loss of reduction requiring later surgical interventions (Table 6).

**Table 6.** Ankle Fracture Outcomes: Operative vs Nonoperative Treatment

Investigators	Sample Size	Findings	Miscellaneous
Elgayar et al., 2019	951 closed	Risk of malunion, nonunion, and loss of reduction were greater with nonoperative care	Systematic Review; 5 RCTs surgical vs conservative intervention
Javed et al., 2020	1153 displaced or unstable	No difference in ankle function scores at 6 or 12 months; surgery had lower rates of early tx failure, malunion, and nonunion	Systematic review and Meta-analysis from 7 trials; surgical vs conservative management
Donken et al., 2012	292	Insufficient evidence to conclude whether surgical or conservative treatment produces superior long-term outcomes	Cochrane Database systemic review; surgical vs conservative intervention; 3 RCTs and 1 quasi-RCT
Petrisor et al., 2006	1394 displaced	No significant differences in adverse events or function for operative vs non-operative management	Meta-analysis of 25 RCTs surgical vs conservative intervention

Stress radiographs should be obtained to evaluate for ankle instability secondary to disruption of the syndesmosis or deltoid ligament.^62^ Patient age should not be a determinant when deciding on operative versus nonoperative treatment in the absence of systemic comorbidities such as cardiovascular disease, pulmonary disease, and poorly controlled diabetes with end organ damage.^63^ 2019 systematic review and meta-analysis found that conservative treatment of ankle fractures led to a lower rate of infection, decreased need for further surgery, and improved cost-effectiveness.^57^ Despite these findings, closed reduction often failed, and a large percentage of patients were transitioned to the operative group. The rates of malunion (15%) and nonunion (10%) were also higher in the nonoperative group.^64^

Overall, there have been study findings that ORIF of ankle fractures versus non-operative interventions has provided equivalent functional outcomes.^65^ However, ORIF has also resulted in an anatomic reduction, fewer malunions, and fewer nonunions. Due to the small number of studies, large time range, and heterogeneity between study protocols, no definitive conclusions could be made by the authors.^66,67^

## CONCLUSIONS

Based on this literature review, the authors conclude that nonsurgical treatments for a wide variety of closed extremity fractures can be frequently applied with minimal patient risks. It remains important for orthopedic surgeons to review nonsurgical alternatives for fractures with patients during the decision-making discussions. Less risky and less costly nonoperative methods can sometimes be utilized even with more difficult fractures, particularly with patients possessing higher surgical risks.

### Conflicts of Interest

The authors deny any conflicts of interest.
